# Additivity of Standardized Total Tract Digestible Phosphorus in Mixed Diets and the Influence of Phosphorus Requirement Expressions in Diet Formulations on Phosphorus Excretion in Pigs

**DOI:** 10.3390/ani16010096

**Published:** 2025-12-29

**Authors:** Woong Bi Kwon, Jung Yeol Sung, Hyunsoek Do, Sungkwon Park, Beob Gyun Kim

**Affiliations:** 1 Furst-McNess Company, Rockford, IL 61108, USA; woongbi.kwon@mcness.com; 2 Department of Animal Science, Konkuk University, Seoul 05029, Republic of Korea; jungyeolsung@gmail.com (J.Y.S.); dohyunseok1234@konkuk.ac.kr (H.D.); 3 Department of Food Science and Biotechnology, Sejong University, Seoul 05006, Republic of Korea; sungkwonpark@sejong.ac.kr

**Keywords:** additivity, digestibility, digestible phosphorus, swine

## Abstract

This study aimed to test the additivity of digestible phosphorus (P) in mixed diets fed to pigs and to investigate the effects of P expressions in diet formulations on P excretion from pigs. In experiment 1, eight castrated male pigs (30.5 ± 1.5 kg) were allotted to a replicated 4 × 4 Latin square design. Three experimental diets contained wheat, soybean meal, or both as the sole P source, and a P-free diet was prepared to determine the basal endogenous losses of P. In experiment 2, twenty-four castrated male pigs (18.1 ± 0.7 kg) were allotted to a completely randomized design. Three experimental diets were prepared based on the total, apparent total tract digestible (ATTD), or standardized total tract digestible (STTD) P. In experiment 1, the measured ATTD of P in the mixed diet was greater (*p* < 0.05) than the predicted values, but no difference was observed in the STTD of P in the mixed diet. In experiment 2, the daily P output of the pigs fed the diet based on ATTD P was greater (*p* < 0.05) than that based on the total or STTD P. Overall, the STTD P provides a more accurate and additive measure of biologically available P in mixed diets for pigs, and the use of STTD P in formulations may reduce P excretions.

## Simple Summary

Phosphorus is a key nutrient for bone development and biochemical reactions. An accurate estimation of phosphorus bioavailability is essential to meet the physiological needs of pigs. The apparent total tract digestibility (ATTD) of phosphorus is calculated based on the intake and fecal output of phosphorus in pigs, and the standardized total tract digestibility (STTD) of phosphorus additionally accounts for basal endogenous losses of phosphorus. In this study, we tested the additivity of digestible phosphorus expressions in mixed diets containing wheat and soybean meal fed to pigs, and we compared phosphorus excretions among swine diets formulated based on total, ATTD, or STTD phosphorus. The measured ATTD of phosphorus in the mixed diet was greater than the predicted value, indicating that ATTD phosphorus values were not additive. However, the measured and predicted STTD values did not differ, demonstrating that the STTD phosphorus values are additive across the ingredients. Pigs fed diets formulated based on ATTD phosphorus excreted more phosphorus than those fed diets formulated based on total phosphorus or STTD phosphorus. Overall, the present results indicate that STTD phosphorus provides a more accurate and additive measure of biologically available phosphorus in mixed diets, and the use of STTD phosphorus for diet formulations may reduce phosphorus excretion from pigs.

## 1. Introduction

Phosphorus (P) is a key nutrient in pig nutrition, which is necessary for proper bone development and for regulating biochemical reactions within the body [1,2]. Therefore, pigs should be provided with adequate P to meet their physiological needs, and the accurate estimation of P bioavailability and requirements is essential to achieve this [3]. In the past, swine diets had been formulated based on the relative bioavailability of P calculated using the slope/ratio technique [4,5]. However, because these values are expressed as relative to standard P sources, such as monosodium phosphate, they are not considered additive in mixed diets [6]. This potential violation of the additivity assumption raises concerns about the accuracy of feed formulation because the additivity implies that the amount of digestible nutrients in a mixed diet equals the sum of the digestible nutrients that are contributed by each feed ingredient [7]. Moreover, the bioavailability of the standard P source may not always be 100% because the bioavailability of the reference mineral varies among different sources [8]. Subsequently, the P availability of an ingredient may differ among the studies due to the different standard P sources. Considering these potential limitations, the relative bioavailability of P may not be an ideal basis for formulating swine diets, although it accounts for post-absorptive metabolism [9].

As an alternative to the relative bioavailability of P, digestible total tract P has been commonly used when formulating swine diets [10]. In contrast to a total P basis, digestible total tract P accounts for the digestibility of P in feed ingredients and can be expressed on an apparent (ATTD), true (TTTD), or standardized total tract digestible (STTD) basis [11]. However, ATTD values are not corrected for endogenous losses of P. Consequently, ATTD values would not be expected to be additive in mixed diets, particularly when the ingredients used in the mixed diets have very low P concentrations [12]. This is consistent with the non-additivity reported in diets formulated based on apparent ileal digestible amino acids [13,14]. In contrast to ATTD, TTTD values would be expected to be additive in mixed diets because the TTTD values account for total endogenous P losses, including both basal and specific endogenous losses [6,15]. However, the descriptive term of true total tract digestibility has been used inconsistently: it may refer either to the digestibility estimated by the regression method or to the digestibility that accounts for total endogenous losses measured using the homoarginine or isotope tracer dilution methods [16]. Regardless, true digestibility is laborious and costly to measure, which limits its application in swine diet formulation [17]. The STTD basis occupies an intermediate position between ATTD and TTTD because it only accounts for basal endogenous losses (BEL) of P, which can be easily measured using a P-free diet [18,19]. In addition, STTD values are expected to be additive in mixed diets regardless of the P concentration in feed ingredients [12], similar to the additivity observed in mixed diets formulated based on standardized ileal digestible amino acids [13,14].

In the current NRC [10], P requirements are provided on total, ATTD, and STTD bases. She et al. [12] reported that values for STTD of P in corn, soybean meal (SBM), and canola meal were additive when these ingredients were included in mixed diets fed to 21 kg pigs. However, the BEL of P was not measured in She et al. [12]; instead, a value of 190 mg/kg dry matter intake (DMI), provided by NRC [10], was used. Although the BEL of P (139 to 252 mg/kg DMI) measured using a P-free diet are relatively consistent across different studies [18,20], measuring the BEL of P is recommended when testing the additivity of P. It is also valuable to validate whether STTD values are additive in wheat-based diets, rather than corn-based diets, to demonstrate that their additivity is independent of diet type. Technically, P excretion is expected to be lower when swine diets are formulated based on STTD P rather than total or ATTD P because STTD P considers the P digestibility of ingredients as well as the BEL of P. However, it remains unclear whether formulating swine diets based on an STTD P basis according to the NRC [10] significantly reduces fecal P excretion compared with formulations based on a total or ATTD P basis. Therefore, the objective of this study was to test the hypotheses that formulating diets based on STTD P is additive in mixed diets and reduces fecal P excretion compared with formulations based on total or ATTD P in growing pigs.

## 2. Materials and Methods

The procedures for the animal experiment were approved by the Institutional Animal Care and Use Committee of Konkuk University (Seoul, Republic of Korea, KU14094 and KU15034).

### 2.1. Animals, Diets, and Experimental Design

In experiment 1, eight crossbred castrated male pigs (Landrace × Yorkshire × Duroc) with an initial body weight (BW) of 30.5 kg (standard deviation = 1.5) were used to determine the additivity of ATTD of P and STTD of P in a mixed diet containing wheat and SBM as the sole P sources (Table 1).

Four experimental diets were prepared (Table 2). Two diets were formulated to contain either wheat (70.0%) or SBM (50.0%) as the sole source of dietary P in each diet. The third diet contained both wheat (55.7%) and SBM (30.6%) at the expense of cornstarch. A P-free diet was also formulated, mainly based on cornstarch, gelatin, and sucrose, to measure the BEL of P from the pigs. All experimental diets were formulated to maintain the constant total calcium-to-total P ratio of 1.5:1 to 1.6:1 except for the P-free diet (Table 3). The animals were allotted to a replicated 4 × 4 Latin square design with 4 dietary treatments and 4 periods [21] to obtain 8 observations for each dietary treatment. The animals were individually housed in metabolism crates equipped with a feeder.

In experiment 2, twenty-four castrated male pigs (Landrace × Yorkshire × Duroc) with an initial BW of 18.1 kg (standard deviation = 0.7) were used to test the hypothesis that formulating diets based on STTD P reduces fecal P excretion compared with formulations based on total or ATTD P in growing pigs. Corn, SBM, dried whey powder, rice bran, and dicalcium phosphate were used as P sources in the experimental diets (Table 4). Three experimental diets were prepared based on the total P, ATTD P, or STTD P to meet the P requirement estimates by adjusting the inclusion rate of rice bran at the expense of cornstarch and cellulose (Table 5). Rice bran was used as a source of P with a low ATTD of P (13%) and STTD of P (23%) in the experimental diets based on the NRC [10]. Vitamins and minerals were included in all diets in experiments 1 and 2 to meet or exceed the nutrient requirement estimates [10]. The pigs were randomly allotted to the 3 dietary treatments with 8 replicates per diet in a completely randomized design. The pigs were individually housed in metabolism crates that were equipped with a feeder.

### 2.2. Feeding and Sample Collection

The amount of feed provided daily per pig was calculated as approximately 3 times the estimated maintenance requirement for metabolizable energy (i.e., 197 kcal/kg of BW^0.60^; NRC [10]). The daily feed allowance was divided into 2 equal meals and fed to pigs at 0800 and 1600 h. The feed allowance for each pig was adjusted by reflecting the BW of the pigs at the beginning of each period. Water was freely accessible at all times. An experimental period consisted of a 4-day adaptation period and a 5-day collection period. Feces were collected according to the marker-to-marker procedure [22] using ferric oxide as an indigestible marker. Ferric oxide was added to the morning diets on days 5 and 10. The collected feces were immediately stored at −20 °C in the freezer.

### 2.3. Chemical Analysis

The frozen feces were dried in a forced-air drying oven at 55 °C, ground, and mixed before analysis. Gross energy in the diet samples was determined using bomb calorimetry (Parr 1261; Parr Instruments Co., Moline, IL, USA). Samples of the ingredients and diets were analyzed for dry matter (method 930.15), crude protein (method 990.03), ether extract (method 920.39), crude fiber (method 978.10), neutral detergent fiber (method 2002.04), acid detergent fiber (method 973.18), and ash (method 942.05) according to the AOAC [23]. Samples of ingredients, diets, and feces were digested by the wet digestion method in the presence of perchloric acid (method 957.02), and the P and calcium concentrations were determined by using an inductively coupled plasma spectroscopy (method 985.01) according to the AOAC [23].

### 2.4. Calculations

In experiment 1, the ATTD of P in the experimental diets was calculated based on the P intake and the amount of P excretion as follows [18]:

ATTD of P (%) = (P_i_ − P_o_) ÷ P_i_ × 100

where P_i_ is the total P intake (g) during the collection period, and P_o_ is the total fecal P output (g) during the collection period.

The ATTD of P in wheat- or SBM-containing diets was regarded as the values of ATTD of P in each ingredient because each test ingredient was the only source of P in the diets. The relative contributions of P from wheat and SBM used in the mixed diet were calculated based on the analyzed P contents in wheat and SBM, respectively, and used for the calculation of the predicted ATTD and STTD of P values in the mixed diet (Table 3).

The predicted ATTD of P in the mixed diet was calculated based on the values of ATTD of P in wheat and SBM according to the following equation [24]:

Predicted ATTD of P (%) in a mixed diet = [(P_W_ × ATTD_W_) + (P_SBM_ × ATTD_SBM_)] ÷ (P_W_ + P_SBM_)

where P_W_ and P_SBM_ represent the P concentrations (%) in the mixed diet in which wheat and SBM are the sole sources of P, respectively; and ATTD_W_ and ATTD_SBM_ represent the measured ATTD of P (%) in wheat and SBM, respectively.

The BEL of P was determined based on the DMI (kg) and the amount of P excretion from the pigs fed the P-free diet. To determine the STTD of P, the ATTD of P was then corrected by the BEL of P (mg/kg DMI), and the STTD of P in diets was calculated according to the following equation [18]:

STTD of P (%) = [P_i_ − (P_o_ − BEL of P × DMI × 1000) ÷ P_i_] × 100

where P_i_ is the total P intake (g) during the collection period, and P_o_ is the total fecal P output (g) during the collection period. The predicted STTD of P in the mixed diet was calculated based on the values of measured STTD of P in wheat and SBM according to the following equation:

Predicted STTD of P (%) in a mixed diet = [(P_W_ × STTD_W_) + (P_SBM_ × STTD_SBM_)] ÷ (P_W_ + P_SBM_)

where STTD_W_ and STTD_SBM_ represent the measured STTD of P (%) in wheat and SBM, respectively. The ATTD and STTD of P for each ingredient that was determined from the same pig were used to calculate the predicted ATTD and STTD of P in the mixed diet.

In experiment 2, the ATTD of P (%) in the diets was calculated using the same method as in experiment 1.

### 2.5. Statistical Analysis

In experiment 1, data were analyzed using the MIXED procedure of SAS version 9.4 (SAS Inst. Inc., Cary, NC, USA). The experimental unit was a pig, and the model included dietary treatment as a fixed variable, replication, animal within replication, and period within replication as random variables. The least squares mean of each dietary treatment was calculated, and the differences between means were tested using the PDIFF option with Tukey’s adjustment [25]. The difference between measured and predicted digestibility values of P in the mixed diet was compared using a paired t-test. In experiment 2, data were analyzed using the GLM procedure of SAS. The model included dietary treatment as an independent variable. The procedures for each treatment mean calculation and pairwise comparisons among the dietary treatment groups were the same as in experiment 1. In both experiments, the experimental unit was a pig, and the statistical significance was determined by an alpha level of 0.05.

## 3. Results

### 3.1. Experiment 1

All animals were healthy and readily consumed the provided feed throughout the experimental period. The values for the ATTD of P in the mixed diet containing wheat and SBM were greater (*p* < 0.05) than in SBM (Table 6).

In the present study, the difference between the measured and predicted values for the ATTD and STTD of P in the mixed diet was compared (Table 7). The measured ATTD of P in the mixed diet was greater (45.1% vs. 41.3%; *p* = 0.046) than the predicted ATTD of P. However, there was no difference (*p* = 0.258) between the measured and predicted values for STTD of P in the mixed diet.

### 3.2. Experiment 2

All animals were healthy and readily consumed the provided feeds throughout the experimental period. During the collection period, the total feed intake did not differ among the treatments (Table 8). However, the daily P intake of pigs fed the diet based on the ATTD P basis was greater (10.9 vs. 7.65 or 8.96 g/d, respectively; *p* < 0.05) compared with the total P- or STTD P-based group. The daily fecal P output of the pigs fed the diet based on ATTD P was greater (4.27 vs. 2.60 or 3.30 g/d, respectively; *p* < 0.05) than the diet based on the total P or STTD P basis.

## 4. Discussion

### 4.1. Experiment 1

The P content in the wheat used in the present study was similar to the value reported by Sauvant et al. [26], but slightly less than the value reported by the NRC [10]. The P content in SBM used in the present study was within the range of previously reported values [10,26,27,28,29]. The ATTD of P in wheat in the present work was slightly less than the values reported in the literature [10,26,30]. The ATTD of P in SBM in the present study was very similar to the values in the literature [10,18,26,27,30]. The determined BEL of P in the pigs fed the P-free diet in the present study was 198 ± 58 mg/kg DMI, which is in agreement with the values provided by previous studies (139 to 252 mg/kg DMI) [18,20] and the NRC [10]. In contrast to the ATTD of P, the values for the STTD of P in the SBM and mixed diet were not different, which would be explained by the contribution of the BEL of P to the total fecal output of P. Considering the lower dietary P concentration and ATTD of P in the SBM diet, the contribution of the BEL of P to the total fecal output of P would be expected to be greater in the SBM diet compared with the mixed diet, leading to a greater increase from ATTD to STTD of P in the SBM diet. The STTD of P in wheat determined in this experiment was less than the value (49.3% vs. 56.0%) reported by the NRC [10]. However, the value for the STTD of P in wheat reported by the NRC [10] was based on only two studies. Therefore, more studies are needed to obtain more reliable STTD of P in wheat. The STTD of P in SBM is in good agreement with the previously reported values [18,27].

Based on the different ATTD of P between the measured and predicted values, the ATTD values of P are not additive. However, the lack of a significant difference between measured and predicted STTD of P indicates that the STTD values of P are additive in the mixed diet. However, both the measured ATTD and STTD values of P were numerically greater than the predicted values. This observation is likely due to the fact that the wheat contains an intrinsic phytase enzyme and has a relatively high phytase activity compared to other cereal grains [31,32]. The intrinsic phytase in a feed ingredient having high phytase activity can promote not only the degradation of its phytate but also the degradation of phytate in other ingredients [26,33]. It should be noted, however, that the intrinsic phytase activity in feed ingredients of plant origin may be inactivated during feed processing such as drying, granulating, or extracting at temperatures above 80 °C [31,33]. Therefore, it is necessary to know two values for P digestibility in feed ingredients with or without heat processing for the additivity of digestible P concentrations in mixed diets when using feed ingredients with a significant intrinsic phytase activity, such as wheat, rye, barley, triticale, and their co-products [26].

The studies on the additivity of digestible P in mixed diets for swine are very limited, but they have previously been reported by Fang et al. [34], who determined the additivity of the ATTD or TTTD of P in two different mixed diets containing five ingredients for growing pigs. Fang et al. [34] demonstrated that the ATTD of P values are not always additive, whereas the TTTD of P values are additive when using ingredients containing low levels of phytate P and anti-nutritional factors such as tannin, hemagglutinin, and trypsin in swine diets. Similarly, She et al. [12] also reported that the ATTD of P in a mixture of corn, SBM, and canola meal was not additive for 20-kg pigs. However, another previous study showed that the ATTD of P in SBM and wheat was additive in a mixture of SBM and wheat when microbial phytase was not used in the experimental diets [29]. The reason for this discrepancy is unclear, but the source of wheat may have affected the additivity of digestible P in wheat–SBM mixed diets. The ATTD of P in four sources of wheat ranged from 61% to 74% [29], likely due to varying intrinsic phytase activities. Apparently, the phytase in feed ingredients increases P digestibility in the ingredients and mixed diets [27]. The potentially large quantity of intrinsic phytase in the wheat in the previous experiment [29] may have resulted in additive ATTD P in the wheat–SBM mixed diet.

It is believed that the diet formulation based on the values for the STTD of P was considered more additive in mixed diets than on the values for the ATTD of P because the STTD values were calculated by correcting the ATTD values for the BEL of P [35]. According to the results of the current study, the ATTD values of P are not additive, whereas the STTD values of P are additive in the SBM–wheat-based diet. The ATTD values of P are also largely variable within the same feed ingredients [36,37]. Therefore, the STTD values of P should be used in diet formulations for the additivity of P in mixed diets for pigs.

### 4.2. Experiment 2

The reason for the difference in daily P intake of pigs among treatments is most likely due to the different P contents in the experimental diets, as the daily feed intake was comparable among the experimental diets. The increased P intake led to increased fecal P excretion in pigs that were fed the diet formulated based on ATTD P, as ATTD of P did not differ among the experimental diets.

Rice bran is a non-conventional feed ingredient for swine diets, but it is an available feed ingredient as a source of energy [37,38]. Among the plant origin feed ingredients available for swine listed by the NRC [10], rice bran is the ingredient that contains the greatest amount of total P (2.16%), but approximately 80% of the P in rice bran is bound to phytic acid, which prohibits the digestion of P. According to the NRC [10], the ATTD of P and STTD of P in rice bran are 0.13% and 0.23%, respectively. The low P digestibility in rice bran is likely due to the large quantity of phytate P. In the current study, rice bran was used in experimental diets to meet the total P, ATTD P, and STTD P requirement estimates by adjusting the inclusion rate based on the digestibility of P in the rice bran suggested by the NRC [10].

The requirement estimate of total P, ATTD P, and STTD P for 11 to 25-kg pigs is 0.60%, 0.29%, and 0.33%, respectively [10]. The P requirement, based on total P, ATTD P, or STTD P, was determined using a corn–SBM-based diet, which is a standard diet for the requirement estimates [10]. Thus, the use of rice bran in the current study led to a shifted level of total dietary P in the experimental diets, depending on the different formulation methods. As a result, the analyzed P concentrations in the experimental diets ranged from 0.76% to 1.09%.

Formulating diets based on the ATTD P with rice bran, which is less digestible than a corn-SBM-based diet, may have led to an oversupply of P to pigs because the ATTD-based P contents in feed ingredients are not additive in mixed diets [18]. Therefore, it is believed that formulating diets based on the STTD P of feed ingredients can marginally meet the P requirement of pigs without an oversupply of P. On the other hand, formulating diets based on the total P can lead to an undersupply of P to pigs because total P is not the value considering any biological aspects, such as the availability or digestibility of P for pigs.

Taken together, the results from this experiment indicate that formulating a diet based on a total P or STTD P basis resulted in less fecal P excretion compared with the diet based on an ATTD P basis. However, it still remains unclear whether formulating diets based on the three different P requirement estimates affects urinary P excretion. Therefore, further research is required to investigate whether formulating diets based on the three different P requirement estimates may influence growth performance, and to confirm P retention in pigs because a surplus of P will be excreted into the urine via the kidney [39].

## 5. Conclusions

Overall, the apparent total tract digestibility values of phosphorus are not additive, whereas standardized total tract digestibility values of phosphorus may be additive when formulating swine diets. In addition, formulating a diet based on total phosphorus or standardized total tract digestible phosphorus resulted in less phosphorus excretion, mainly due to the lower concentration of dietary phosphorus compared with the diet that was based on the apparent total tract digestible phosphorus. Further research is warranted to investigate growth performance and phosphorus retention in pigs fed diets formulated based on total phosphorus, apparent total tract digestible phosphorus, or standardized total tract digestible phosphorus.

## Figures and Tables

**Table 1:** Energy and nutrient composition of wheat and soybean meal (as-is basis; experiment 1) ^1^.

Item	Wheat	Soybean Meal
Dry matter, %	89.3	90.3
Gross energy, kcal/kg	4007	4199
Crude protein, %	12.72	42.57
Ether extract, %	2.50	1.80
Crude fiber, %	1.86	5.33
Ash, %	1.88	5.41
Phosphorus, %	0.15	0.45
Calcium, %	0.34	0.68
Neutral detergent fiber, %	9.33	10.50
Acid detergent fiber, %	2.70	6.41

^1^ Means of duplicate analyses of each ingredient.

**Table 2:** Ingredient composition of experimental diets (as-fed basis; experiment 1).

Item, %	Wheat	Soybean Meal	Wheat–Soybean Meal	Phosphorus-Free
Wheat	70.0	-	55.7	-
Soybean meal, 48% crude protein	-	50.0	30.6	-
Cornstarch	16.6	36.7	-	45.6
Sucrose	10.0	10.0	10.0	20.0
Soybean oil	2.00	2.00	2.00	4.00
Gelatin	-	-	-	23.0
Cellulose	-	-	-	4.00
_L_-Lysine∙HCl, 78.8%	-	-	-	0.22
_DL_-Methionine, 99.0%	-	-	-	0.08
_L_-Threonine, 98.5%	-	-	-	0.20
_L_-Tryptophan, 98.0%	-	-	-	0.16
_L_-Histidine, 98.5%	-	-	-	0.20
_L_-Isoleucine, 98.5%	-	-	-	0.24
_L_-Valine, 98.0%	-	-	-	0.18
Limestone	0.70	0.60	1.00	0.96
Potassium carbonate	-	-	-	0.40
Magnesium oxide	-	-	-	0.08
Salt	0.40	0.40	0.40	0.40
Vitamin-mineral premix ^1^	0.30	0.30	0.30	0.30

^1^ Provided the following quantities per kg of complete diet: vitamin A, 25,000 IU; vitamin D_3_, 4000 IU; vitamin E, 50 IU; vitamin K, 5.0 mg; thiamin, 4.9 mg; riboflavin, 10.0 mg; pyridoxine, 4.9 mg; vitamin B_12_, 0.06 mg; pantothenic acid, 37.5 mg; folic acid, 1.10 mg; niacin, 62 mg; biotin, 0.06 mg; Cu, 25 mg as copper sulfate; Fe, 268 mg as iron sulfate; I, 5.0 mg as potassium iodate; Mn, 125 mg as manganese sulfate; Se, 0.38 mg as sodium selenite; Zn, 313 mg as zinc oxide; butylated hydroxytoluene, 50 mg.

**Table 3:** Chemical composition of experimental diet (as-fed basis; experiment 1) ^1^.

Item	Wheat	SBM	Wheat–SBM	P-Free
Dry matter, %	92.1	92.7	92.3	93.2
Gross energy, kcal/kg	4027	4087	4079	4206
Crude protein, %	9.03	21.6	19.3	24.5
Ether extract, %	3.16	2.60	3.50	4.02
Crude fiber, %	1.52	3.07	3.20	1.93
Ash, %	2.33	3.89	3.87	1.66
Phosphorus, %	0.39	0.50	0.61	0.01
Calcium, %	0.24	0.34	0.40	0.37
Neutral detergent fiber, %	6.01	4.86	7.92	2.61
Acid detergent fiber, %	2.02	3.26	3.46	1.35
Relative contribution of phosphorus ^2^, %				
Total	100.0	100.0	100.0	-
Wheat	100.0	-	48.1	-
Soybean meal	-	100.0	51.9	-

P-free = phosphorus-free diet; SBM = soybean meal-based diet; Wheat = wheat-based diet; Wheat–SBM = wheat–soybean meal-based diet. ^1^ Means of duplicate analyses of each ingredient. ^2^ Relative contribution of phosphorus, % = phosphorus contribution from corresponding ingredients (g) ÷ phosphorus content in the mixed diet (g) × 100.

**Table 4:** Analyzed phosphorus concentration in corn, soybean meal, whey powder, rice bran, and dicalcium phosphate (as-is basis; experiment 2) ^1^.

Item	Phosphorus, %
Corn	0.28
Soybean meal	0.61
Dried whey powder	0.81
Rice bran	1.88
Dicalcium phosphate	19.3

^1^ Means of duplicate analyses of each ingredient.

**Table 5:** Ingredient composition and chemical composition of experimental diets (as-fed basis; experiment 2).

Item	Total P	ATTD P	STTD P
Ingredient, %			
Ground corn	38.9	38.9	38.9
Soybean meal, 48% crude protein	25.0	25.0	25.0
Dried whey powder	10.0	10.0	10.0
Rice bran	6.30	20.0	11.7
Cornstarch	9.96	-	6.01
Cellulose	3.60	-	2.22
Soybean oil	3.00	3.00	3.00
Limestone	1.00	1.00	1.00
Dicalcium phosphate	0.60	0.60	0.60
_L_-Lysine∙HCl, 78.8%	0.48	0.39	0.45
_DL_-Methionine, 99.0%	0.20	0.15	0.18
Salt	0.50	0.50	0.50
Vitamin-mineral premix ^1^	0.50	0.50	0.50
Chemical composition ^2^			
Dry matter, %	90.7	90.3	90.7
Gross energy, kcal/kg	4151	4283	4237
Crude protein, %	17.7	18.9	17.4
Ether extract, %	5.57	7.77	6.06
Crude fiber, %	3.92	3.59	3.85
Ash, %	4.95	5.85	5.50
Phosphorus, %	0.76	1.09	0.91
Calcium, %	0.86	0.80	0.91
Neutral detergent fiber, %	8.68	9.26	9.24
Acid detergent fiber, %	5.53	4.14	5.11

ATTD P = a diet formulated based on apparent total tract digestible phosphorus; STTD P = a diet formulated based on standardized total tract digestible phosphorus; Total P = a diet formulated based on total phosphorus. ^1^ Provided the following quantities per kg of complete diet: vitamin A, 25,000 IU; vitamin D_3_, 4000 IU; vitamin E, 50 IU; vitamin K, 5.0 mg; thiamin, 4.9 mg; riboflavin, 10.0 mg; pyridoxine, 4.9 mg; vitamin B_12_, 0.06 mg; pantothenic acid, 37.5 mg; folic acid, 1.10 mg; niacin, 62 mg; biotin, 0.06 mg; Cu, 25 mg as copper sulfate; Fe, 268 mg as iron sulfate; I, 5.0 mg as potassium iodate; Mn, 125 mg as manganese sulfate; Se, 0.38 mg as sodium selenite; Zn, 313 mg as zinc oxide; and butylated hydroxytoluene, 50 mg. ^2^ Means of duplicate analyses of each experimental diet.

**Table 6:** Digestibility of phosphorus (P) in experimental diets fed to growing pigs (experiment 1) ^1^.

Item	Wheat	SBM	Wheat–SBM	SEM	*p*-Value
Feed intake					
Total feed intake, g/d	1152 ^a^	1055 ^b^	1167 ^a^	42	0.001
P intake, g/d	2.78 ^c^	3.57 ^b^	4.65 ^a^	0.14	<0.001
Fecal output					
Total feces, g/d	84 ^b^	60 ^c^	105 ^a^	5	<0.001
P in feces, %	1.94 ^c^	3.51 ^a^	2.45 ^b^	0.08	<0.001
P output, g/d	1.62 ^c^	2.12 ^b^	2.55 ^a^	0.10	<0.001
Digestibility, %					
ATTD of P	41.8 ^ab^	40.9 ^b^	45.1 ^a^	1.1	0.034
STTD of P ^2^	49.3	46.4	49.7	1.1	0.085

ATTD = apparent total tract digestibility; SBM = soybean meal-based diet; SEM = standard error of the means; STTD = standardized total tract digestibility; Wheat = wheat-based diet; Wheat–SBM = wheat–soybean meal-based diet. ^a–c^ Within a row means without a common superscript letter differ (*p* < 0.05). ^1^ Each least squares mean represents 8 observations. ^2^ Values for STTD of P were calculated by correcting ATTD values for the basal endogenous losses of P. The basal endogenous losses of P were determined in pigs fed the P-free diet at 198 ± 58 mg/kg dry matter intake.

**Table 7:** Measured and predicted values for apparent total tract digestibility (ATTD) and standardized total tract digestibility (STTD) of phosphorus (P) in a wheat–soybean meal-based diet (experiment 1) ^1^.

Item, %	Measured	Predicted ^2^	Difference ^3^	SE	*p*-Value
ATTD of P	45.1	41.3	3.8	1.6	0.046
STTD of P	49.7	47.8	1.9	1.6	0.258

^1^ Measured and predicted values represent 8 observations. ^2^ Predicted values are calculated based on the determined ATTD or STTD of P for soybean meal and wheat. ^3^ The difference is calculated by subtracting the predicted ATTD or STTD of P from the measured value.

**Table 8:** Digestibility of phosphorus (P) in experimental diets fed to pigs and P excretion of pigs fed experimental diets (as-fed basis; experiment 2) ^1^.

Item	Total P	ATTD P	STTD P	SEM	*p*-Value
Feed intake					
Total feed intake, g/d	1005	994	988	16	0.326
P intake, g/d	7.65 ^c^	10.9 ^a^	8.96 ^b^	0.15	<0.001
Fecal output					
Total feces, g/d	139	151	138	10	0.140
P in feces, %	1.83 ^c^	2.84 ^a^	2.39 ^b^	0.20	<0.001
Fecal P output, g/d	2.60 ^c^	4.27 ^a^	3.30 ^b^	0.16	<0.001
ATTD of P, %	65.8	60.7	63.0	2.2	0.113

ATTD = apparent total tract digestibility; ATTD P = a diet formulated based on an apparent total tract digestible phosphorus; SEM = standard error of the means; STTD P = a diet formulated based on a standardized total tract digestible phosphorus; Total P = a diet formulated based on a total phosphorus. ^a–c^ Within a row means without a common superscript letter differ (*p* < 0.05). ^1^ Each least squares mean represents 8 observations.

## Data Availability

The data presented in the manuscript have been read and agreed to by the authors for the published version of the manuscript.

## Abbreviations

The following abbreviations are used in this manuscript:

ATTD	Apparent total tract digestible
BEL	Basal endogenous losses
BW	Body weight
DMI	Dry matter intake
P	Phosphorus
SBM	Soybean meal
STTD	Standardized total tract digestible
TTTD	True total tract digestible
